# High‐Throughput Nanorheology of Living Cells Powered by Supervised Machine Learning

**DOI:** 10.1002/aisy.202400867

**Published:** 2025-04-15

**Authors:** Jaime R. Tejedor, Ricardo Garcia

**Affiliations:** ^1^ Instituto de Ciencia de Materiales de Madrid CSIC c/Sor Juana Inés de la Cruz 3 28049 Madrid Spain

**Keywords:** atomic force microscopies, mammalian cells, mechanobiologies, nanoindentations, nanorheologies

## Abstract

Atomic force microscopy (AFM) is extensively applied to measure the nanomechanical properties of living cells. Despite its popularity, some applications on mechanobiology are limited by the low throughput of the technique. Currently, the analysis of AFM‐nanoindentation data is performed by model fitting. Model fitting is slow, data intensive, and prone to error. Herein, a supervised machine‐learning regressor is developed for transforming AFM force–distance curves into nanorheological behavior. The method reduces the computational time required to process a force volume of a cell made of 2.62 × 10^5^ curves from several hours to minutes. In fact, the regressor increases the throughput by 50‐fold. The training and the validation of the regressor are performed by using theoretical curves derived from a contact mechanics model that combined power–law rheology with bottom effect corrections and functional data analysis. The regressor predicts the modulus and the fluidity coefficient of mammalian cells with a relative error below 4%.

## Graphical abstract

Herein, atomic force microscopy (AFM) and machine learning are combined to determine the viscoelastic properties of living cells at the nanoscale. A universal regressor that predicts the viscoelastic properties of mammalian cells from AFM experiments is developed. The regressor predicts the viscoelastic parameters of two cell lines. The regressor outperforms state‐of‐the art model fitting methods by a factor 50 in speed.

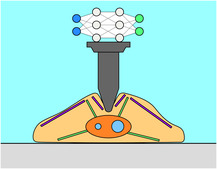

## 1 Introduction

Atomic force microscopy (AFM) methods are applied to measure time‐dependent properties of living cells and tissues.^[^ ^1^, ^2^, ^3^, ^4^ ^]^ Those measurements are central to describe and, eventually, understand the mechanical response of single cells to external forces.^[^ ^5^, ^6^, ^7^, ^8^, ^9^, ^10^, ^11^, ^12^, ^13^, ^14^ ^]^ Viscoelastic property measurements might provide markers of physiology and disease.^[^ ^15^, ^16^, ^17^, ^18^ ^]^ In addition, it has been shown that viscoelastic parameters might be used to enhance imaging contrast and spatial resolution in AFM images.^[^ ^19^ ^]^

A central issue regarding the application of AFM on cell biology is the low throughput. AFM measurements on cells and tissues are slow and time consuming. To consolidate and expand the applications of AFM in mechanobiology and clinical research, speeding up imaging and data analysis is required.

The throughput in AFM is controlled by several factors, which might be grouped in two near‐independent categories, data acquisition and data processing. High‐speed and multifrequency methods have developed solutions to address the limitations associated with data acquisition.^[^ ^20^, ^21^, ^22^ ^]^ In contrast, machine‐learning methods might offer alternatives to reduce data processing time.^[^ ^23^, ^24^, ^25^, ^26^, ^27^, ^28^ ^]^ This contribution addresses the development of a machine‐learning regressor to speed up the transformation of AFM data into viscoelastic or rheological properties.

**Figure 1:**
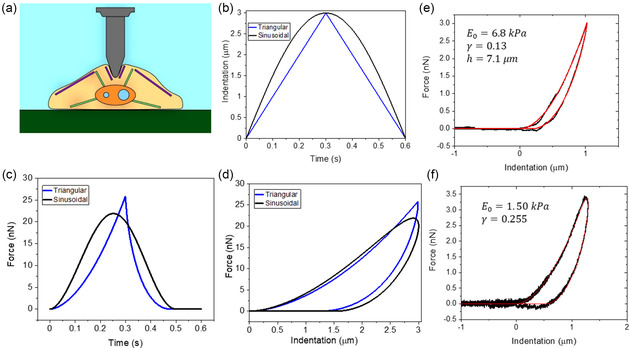
a) Scheme of an AFM‐nanoindentation experiment on a cell. b) Examples of some common indentation profiles: triangular and capped sinusoidal indentation profiles (*v* = 10 μm s^−1^). c) Synthetic (theoretical) force versus time curves for the indentations shown in (b). d) FDCs obtained by combining (b) and (c). The synthetic FDCs were calculated by using a cell with *E*_0_ = 5 kPa, *γ* = 0.3, and *h* = 7 μm. e,f) Experimental FDCs (black) and single power‐law rheology fittings (red). The FDCs were obtained at (e) 10 μm s^−1^ and (f) 300 μm s^−1^ on an HeLa cell (over the nuclear region). The modulus and fluidity parameters are included in the panels. Experimental FDCs by Gisbert et al.^[^ ^14^ ^]^

Several AFM‐based methods have been implemented to study the nanorheology of cells and tissues.^[^ ^1^, ^2^, ^3^ ^]^ Among them, AFM‐based nanoindentation stands outs as the most popular approach to study the relationship between forces, deformation, and mechanical properties of cells and tissues. In this method, a force–distance curve (FDC) is obtained by measuring the deflection of the cantilever‐tip system as a function of the cell's indentation (**Figure** **1**a). In its most common configuration, the motion of the cantilever‐tip system is controlled by a piezo actuator, which follows a triangular or capped sinusoidal signal (Figure 1b). The deflection of the cantilever is transformed into force versus time (Figure 1c) or, more commonly, FDCs (Figure 1d). Relevant mechanical parameters are obtained by fitting the experimental FDCs with the force values given by a contact mechanics model.^[^ ^1^, ^2^ ^]^

Model fitting required knowledge of tip's velocity and geometry, cell thickness, tip‐cell contact point, and baseline effects associated with hydrodynamic drag of the probe displacement. Furthermore, in many situations of interest, model fitting required numerical analysis. As a consequence, the determination of viscoelastic properties at the nanoscale is time consuming, data intensive, and prone to error.^[^ ^29^, ^30^, ^31^ ^]^ Some machine‐learning approaches were applied to improve AFM imaging and classification of single cells.^[^ ^32^, ^33^, ^34^, ^35^, ^36^, ^37^, ^38^ ^]^ However, to our knowledge, machine‐learning regressors have neither been developed nor applied to characterize the nanorheology of living cells. Figure 1e shows some experimental FDCs and the fittings obtained by applying the power‐law rheology model described later.

Herein, we develop a supervised machine‐learning (SML) regressor to process AFM‐based nanoindentation curves. The regressor learns the relationship between nanoindentation data and viscoelastic parameters from theoretical FDCs. Then, it predicts those parameters from experimental FDCs without model fitting. The method has three main steps; synthetic data generation, training, and validation. Training and validation are performed by using sets of theoretical FDCs (synthetic data).

## 2 General Features of the SML Regressor

Two considerations, one of fundamental nature and the other experimental, must be kept in mind to develop a machine‐learning regressor to predict the time‐dependent properties of a cell from AFM data. First, the stress at a given time depends on both the instantaneous deformation and the history of deformation. Second, nonlinear effects of the piezo scanner and/or the noise in the voltage signal applied to the piezo might produce indentation profiles, which depart from ideal triangular or capped sinusoidal waveforms. For those reasons, it is essential to incorporate the history of the deformation in the development of the machine‐learning regressor. This was accomplished by using functional data analysis to generate theoretical data sets to train the regressor. In addition, the regressor was adapted to process time series data. Initially, we considered five different regressor models, convolutional neural networks and four functional neural networks.^[^ ^39^, ^40^ ^]^ Performance tests showed the advantages of using a nested functional basis neural network (nested FBNN). Therefore, the discussion that follows was based on the predictions obtained by the nested FBNN. To simplify the use of acronyms, the aforementioned neural network will be referred as SML. Details of the five neural networks and the performance tests were presented in Supporting Information.

### 2.1 Indentation Profiles

The indentation profiles used during training and validation satisfied the following conditions. The indentation was positive. It started and ended close to zero.

(1a) $$\begin{array}{c} I\left(0\right)=\varepsilon I\left(p\right) \end{array}$$

(1b) $$\begin{array}{c} I\left(1\right)=\varepsilon^{\prime }I\left(p\right) \end{array}$$

*ε* and *ε′* were numbers close to 0. The indentation had a global maximum in the interval (0, 1) which satisfied

(2a) $$\frac{dI}{dt}=0\;(t=p)$$

(2b) $$\begin{array}{c} I\left(p\right)=1 \end{array}$$

The indentation profile was divided into approach and retraction sections, where the derivative was, respectively, positive and negative.

(3) $$\begin{array}{c} \begin{array}{c} \frac{dI(t)}{dt}>0\;\;\text{if}\;t<p\\ \frac{dI(t)}{dt}<0\;\text{if}\;t>p \end{array} \end{array}$$

**Figure 2:**
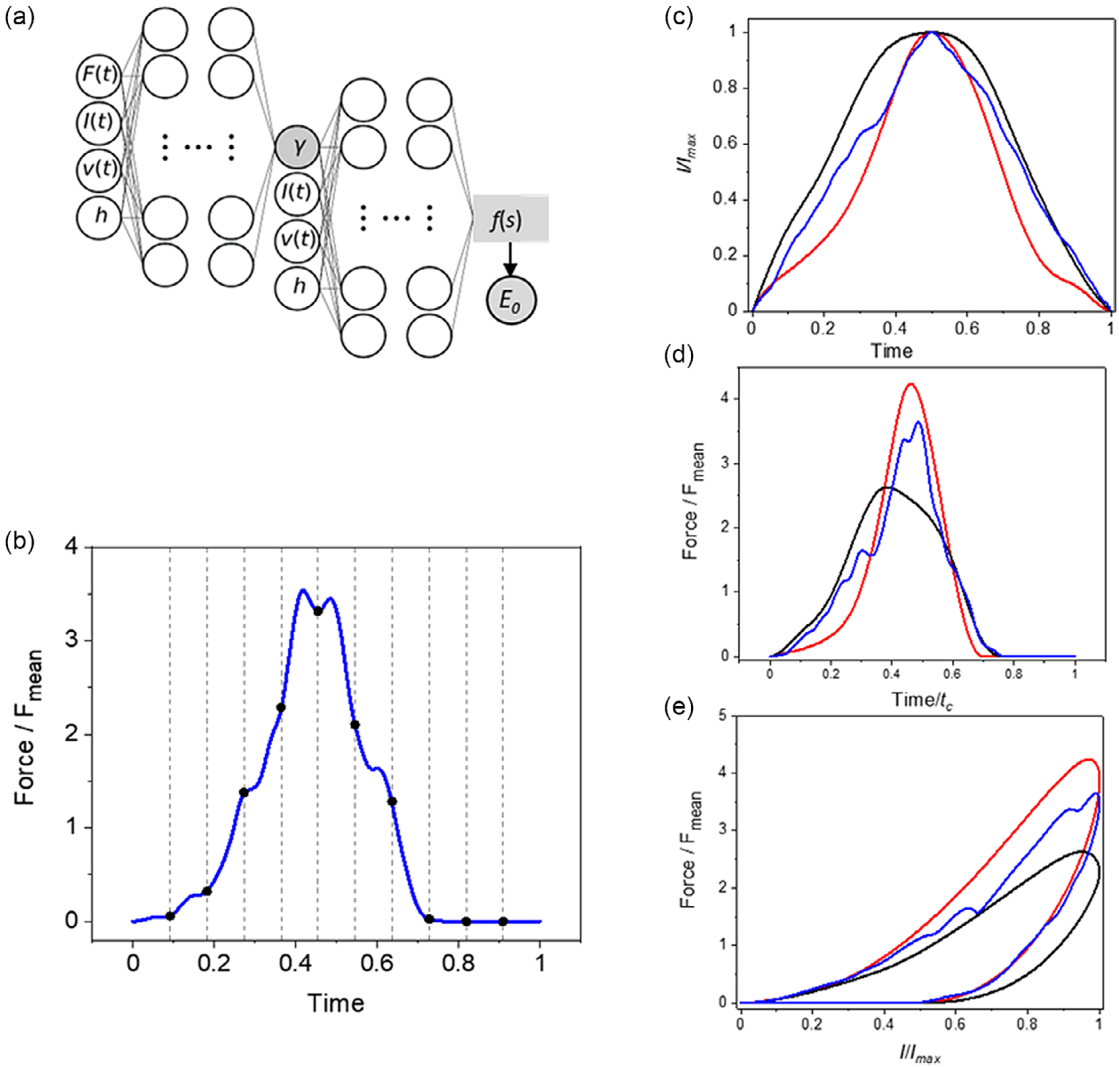
a) Scheme of the SML regressor. The first neural network predicts the fluidity coefficient and the second neural network predicts the mean value of the dimensionless force, *f*(*s*). The modulus is obtained from that value (Equation 15). b) The force is uniformly sampled in the time domain to provide a discretized set of input values. The regressor is trained by sampling 100 time points and a base of splines with 40 knots and degree 3. c) Some examples of the indentation profiles used in training. d) Force–time curves for the indentation profiles shown in (c). e) FDCs for the profiles shown in (c). Parameters: *γ* = 0.3, *I*_max_ = 0.5 h, and *p* = 0.5.

In the aforementioned expressions, time and indentation were made dimensionless so their values remain in the (0, 1) interval. The aforementioned conditions included all the indentation profiles used in AFM‐nanoindentation measurements. Specifically, triangular, sinusoidal, and their combinations^[^ ^2^, ^3^, ^30^, ^41^ ^]^ (**Figure** **2**c–e).

The indentation profiles used to train the regressor were generated from a theoretical model (see later). This approach simplified the training process and gave universality to the regressor. The same regressor was used to predict the nanorheological behavior of different cell lines. To generate the data for training, the indentation profiles were represented using basis expansion. We chose B splines of degree 3 as the basis functions to generate the indentation profiles.

(4) $$\begin{array}{c} I\left(t\right)=\sum_{i=0}^{N}c_{i}B_{i,3}\left(x\right) \end{array}$$

#### 2.1.1 Functional Neuron

The incorporation of functional data analysis into a neural network required to implement a functional neuron. The functional neuron replaced the discrete weights found in common neurons with a weight function. The scalar product of the functional input *f*_*i*_ and the weight function *g* was implemented as

(5) $$\begin{array}{c} \left(f_{i},g\right)=\int f_{i}\left(x\right)g\left(x\right)dx \end{array}$$

Basis expansion was used to compute the gradient of the loss function with respect to the weight functions and to perform backpropagation.

(6) $$\begin{array}{c} g\left(x\right)=\sum_{i=0}^{N}g_{i}B_{i}\left(x\right) \end{array}$$

The integral in Equation (5) was computed by using a basis expansion in the functional inputs.

(7) $$\begin{array}{c} f_{i}\left(x\right)=\sum_{j=0}^{N}c_{j}^{\left(i\right)}B_{j}\left(x\right) \end{array}$$

The associated Gram matrix was calculated using basis functions.

(8) $$\begin{array}{c} G_{ij}=\left(B_{i},B_{j}\right)=\int B_{i}\left(x\right)B_{j}\left(x\right)dx \end{array}$$

The aforementioned scalar product is equivalent to the discrete counterpart with the metric given by *G*_*ij*_.

(9) $$\begin{array}{c} \left(f_{i},g\right)=\int f_{i}\left(x\right)g\left(x\right)dx=\sum_{j,k=1}^{N}c_{j}^{\left(i\right)}g_{k}G_{jk} \end{array}$$

Treating the expansion coefficients, *c*_*j*_, as scalar inputs gave more freedom for training. It enabled to incorporate the Gram matrix into the network weights. Furthermore, the coefficients were standardized to facilitate the training of the regressor. This implementation required a preprocessing step of spline interpolation.

A different scheme was applied to process the force. For each force curve, the force values were sampled to a fixed size using a simple linear interpolation and normalized to the mean value of the FDC. The sampling size was optimized as a hyperparameter in the grid search. In addition, the force values from all the FDCs were normalized by using group normalization.

## 3 Nanorheology and Nested Functional Network

### 3.1 Nanorheology Model for Cells

Single power‐law rheology has been extensively applied to describe the response of a living cell to the mechanical deformations exerted in AFM experiment.^[^ ^42^, ^43^, ^44^, ^45^, ^46^, ^47^, ^48^ ^]^ However, to determine with accuracy the nanorheological behavior of a living cell, the relaxation function of single power–law rheology must be combined with bottom‐effect corrections^[^ ^49^ ^]^ and a tip‐cell contact area, which depends on the history of the deformation.^[^ ^48^ ^]^ The bottom‐effect correction is determined by a polynomial function of the ratio between the contact radius and the cell's height in the region of contact.^[^ ^48^, ^49^ ^]^ As a general rule, bottom‐effect corrections should be implemented whenever the FDCs involved indentations above 500 nm.

The following equations described the contact mechanics model used to train the SML regressor.

(10) $$F\left(I\right)=\{\begin{array}{c} \sum_{j}\alpha_{j}\int_{0}^{t}\psi \left(t-t^{\prime }\right)\frac{d}{dt^{\prime }}\left(I^{\beta_{j}}\left(t^{\prime }\right)\right)dt^{\prime }\;t\leq t_{\text{max}}\\ \sum_{j}\alpha_{j}\int_{0}^{t_{1}\left(t\right)}\psi \left(t-t^{\prime }\right)\frac{d}{dt^{\prime }}\left(I^{\beta_{j}}\left(t^{\prime }\right)\right)dt^{\prime }\;\;t>t_{\text{max}} \end{array}$$

where *α* and *β*_*j*_ were coefficients that depended on the geometry of the probe and the thickness of the cell (*h*), *t*_max_ is the time for which indentation reached its maximum value (*I*_max_), $\psi \left(t-t^{\prime }\right)$ is the viscoelastic relaxation function of the material, and *t*_1_(*t*) is the solution to determine the contact area during tip's retraction.^[^ ^34^ ^]^

(11) $$\begin{array}{c} \int_{t_{1}\left(t\right)}^{t}\psi \left(t-t^{\prime }\right)v\left(t^{\prime }\right)dt^{\prime }=0 \end{array}$$

The relaxation function in power–law rheology was given by

(12) $$\begin{array}{c} \psi \left(t\right)=\frac{E_{0}}{\Gamma \left(1-\gamma \right)}{\left(\frac{t}{t_{0}}\right)}^{-\gamma } \end{array}$$

where Γ is the Euler gamma function and *E*_0_ is a scaling factor with units of force divided by area (scaling modulus). It has been identified as the elastic modulus of the material at time *t*_0_ (commonly *t*_0_ = 1 s). A value of the fluidity coefficient *γ* = 0 defines an elastic solid of Young's modulus *E*_0_ while *γ* = 1 indicates a Newtonian viscous liquid with viscosity *η*_e_ *= E*_0_ *t*_0_. The aforementioned viscoelastic model was successfully applied to determine the viscoelasticity of cells at different speeds and indentation scales.^[^ ^14^, ^46^ ^]^

### 3.2 Nested Functional Network Architecture

The final architecture of the regressor contained two‐nested multilayer perceptron networks. The outer network was devoted to predict the fluidity coefficient and the inner network to predict the modulus (Figure 2a). We found that this architecture was very well suited for handling the constitutive parameters of single power–law rheology. The force, the indentation, the velocity, and the cell thickness were the inputs of the outer network. The force was uniformly sampled with respect to time (Figure 2b) whereas the indentation (and velocity) were expressed through a basis expansion.

The predictions of the fluidity coefficient given by the outer network together with the indentation, the velocity, and the cell thickness were the inputs of the second neural network. This neural network determined the mean value of the dimensionless force *f*(*s*), with *s* = *t*/*t*_tot_ the dimensionless time; *t*_tot_ is the duration of the tip's displacement (approach and retraction). A relationship between *f*(*s*) and the modulus is deduced from Equation 10

(13) $$\begin{array}{c} F\left(t\right)=c\;f\left(s\right)=c\sum_{j}\frac{\alpha_{j}}{\Gamma \left(1-\gamma \right)}\int_{0}^{s}{\left(s-s^{\prime }\right)}^{-\gamma }\beta_{j}i^{\beta_{j}-1}\left(s^{\prime }\right)v\left(s^{\prime }\right)ds^{\prime } \end{array}$$

with

(14) $$\begin{array}{c} \bar{f}=\int_{0}^{1}f\left(s\right)ds \end{array}$$

and finally, the modulus for a conical tip of semiangle *θ* was given by

(15) $$\begin{array}{c} E_{0}=\frac{c}{t_{r}^{1-\gamma }\cdot I_{r}^{\beta_{0}-1}\cdot v_{r}}=\frac{\bar{F}}{\bar{f}}\frac{1}{t_{\text{tot}}^{1-\gamma }\cdot I_{\text{max}}^{\beta_{0}-1}\cdot \bar{v}\cdot \tan\theta } \end{array}$$

(16) $$r_{\text{max}}=\tan\theta \cdot I_{\text{max}}$$

where $\bar{F}$ and $\bar{v}$ are, respectively, the mean force and mean velocity of the FDC; *r*_max_ was the maximum radius of contact. All of the variables were rendered dimensionless so that the neural network validity was not limited to a range of dimensional values (Supporting Information).

The regressor was trained with 100 000 FDCs generated by using the contact mechanics model described in Equation (10)–(12). The curves were generated by applying a random sampling of values for *γ*, and the bottom‐effect correction parameter (*r*_max_/*h*) in the interval (0, 1). Those values included elastic solids (*γ* = 0) and Newtonian liquids (*γ* = 1). They encompassed the range of validity of the bottom effect correction. The accuracy of the regressor was assessed by using the mean absolute percentage error for *E*_0_ and the normalized root‐mean‐squared error for *γ* (Supporting Information).

**Figure 3:**
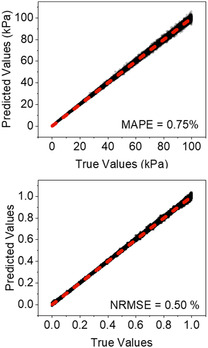
Validation test. Comparison between predicted (regressor) and true (synthetic data). The regressor predicts the modulus and the fluidity coefficient, respectively, with a relative error below 2% and 0.5%. Notice that the same regressor predicts the properties of very stiff (100 kPa) and very soft cells (0.1 kPa). Those values cover the range of values measured on living mammalian cells. The range fluidity coefficient values applied to train the regressor covered all possible values for a material, from an elastic solid (*γ* = 0) to a Newtonian liquid (*γ* = 1).

**Figure** **3** shows the results of a validation test. The values predicted by the regressor were compared to those obtained from synthetic curves (true values). Both curves followed a straight line of slope very close to 1. The accuracy of the regressor was below 1% for both parameters. The same regressor predicted the properties of very stiff cells^[^ ^17^ ^]^ (≈60 – 100 kPa) and very soft cells such as neurons (≈0.1 kPa).^[^ ^50^ ^]^ The aforementioned range (0.1 – 100 kPa) included the expected values for mammalian cells under physiological conditions.

## 4 Application to Mammalian Cells

Next, we applied the SML regressor to predict the viscoelastic parameters of two cell lines (HeLa and fibroblasts [NIH 3T3]). The physical properties and the biological function of those cell lines are different. Fibroblasts are found in many tissues of the human body while HeLa cells are associated with a specific tissue. Fibroblasts are significantly softer than HeLa cells.^[^ ^14^ ^]^ The experimental FDCs were obtained before the development of the SML regressor.^[^ ^14^ ^]^

Therefore, the performance of the regressor was assessed without introducing any bias into the way the experimental data was measured. The results of the comparison will establish the generality of regressors to predict the nanorheology of different cell lines.

**Figure 4:**
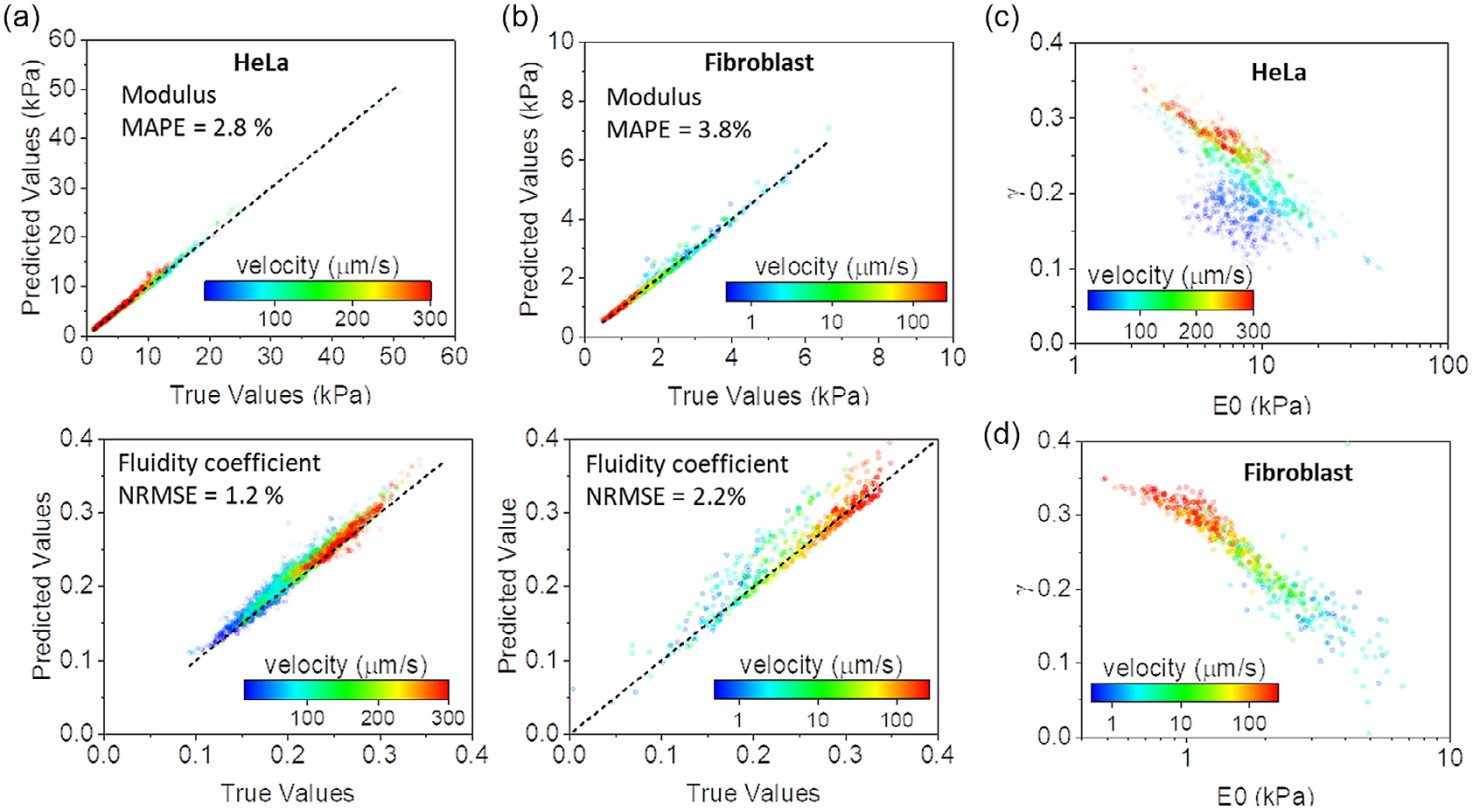
a) Predicted nanorheology of HeLa cells by the SML regressor. b) Predicted nanorheology of an NIH 3T3 fibroblast cell. c) Relation fluidity coefficient and modulus (HeLa). d) Relation fluidity coefficient and modulus (NIH 3T3 fibroblast). The experimental FDCs were obtained by probing the cells at different velocities, for this reason, a color‐code scale bar was included. Experimental FDCs were obtained from an article.^[^ ^14^ ^]^

For the purpose of the comparison, the viscoelastic parameters deduced by applying model fitting were considered the true values of the cell. The AFM data for HeLa cells contained 2400 curves performed on different locations of an HeLa cell (nucleus and cytoplasm) and at different velocities from 10 to 300 μm s^−1^. **Figure** **4**a shows that predicted and true values fell into a straight line of slope close to 1. The values of the modulus and the fluidity coefficient depended on the velocity of the indentation. The modulus decreased as the velocity of the probe was increased from 20 (low *v*) to 1.5 kPa (high *v*). In contrast, the fluidity coefficient increased from 0.1 (low *v*) to 0.35 kPa (high *v)*. The errors for the modulus and the fluidity coefficient were, respectively, 2.8% and 1.2%.

Figure 4b shows the results for fibroblast cells. The modulus decreased as the velocity of the probe was increased from 7 to 0.4 kPa (high velocity). The fluidity coefficient showed an opposite trend. It increased from 0.1 (low velocity) to 0.35 kPa (high velocity). The error for *E*_0_ was below 4% while for *γ* was about 2%.

We remark that the same regressor was used to predict the values of two cell lines (HeLa and fibroblast cell lines) with different biological functions. Therefore, the aforementioned findings were an indication of the generality of the regressor.

The fluidity coefficient and the modulus are not strictly independent parameters.^[^ ^43^, ^51^ ^]^ The fluidity coefficient decreased quasilinearly with the modulus in a semilogarithmic plot. The predictions of the SML regressor showed the aforementioned trend (Figure 4c,d). We noted an increase in the dispersion of the data that for low to medium velocities (>50 μm s^−1^). This effect was quite notorious in the HeLa cells. The underlying mechanism behind the relationship between the fluidity coefficient and the modulus has yet to be clarified.^[^ ^51^ ^]^

### 4.1 High‐Speed Nanorheology Processing

**Figure 5:**
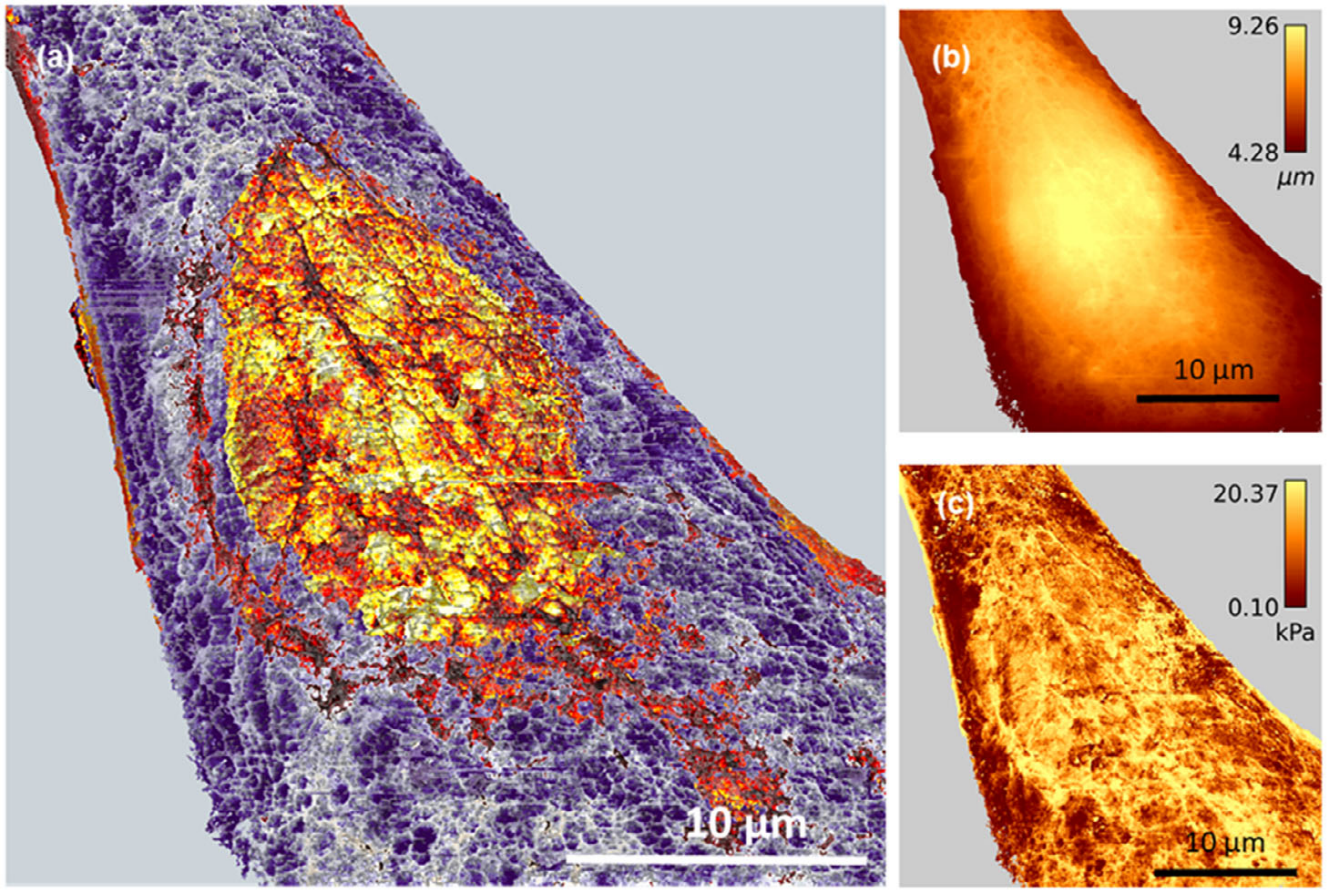
a) High‐spatial resolution compositional map of an HeLa cell powered by machine learning. The map was obtained by applying the regressor to AFM experimental data. The map combined b) topography and c) modulus data. The compositional map shows the fine structure of the actin filament network, the local variation of the modulus, and the size and shape of the nucleus. (b) AFM topography. (c) Scaling modulus map. The final spatial resolution of the regressor map was limited by the number of data points (512 × 512). The experimental FDCs were obtained with a JPK NanoWizard 3.^[^ ^14^ ^]^

To assess the capability of SML to improve the throughput to process AFM data, we compared the time needed to transform force–volume data into a high‐contrast nanomechanical map of a live cell. The map was made by combining in each *xy* location, the modulus and cell's height values.^[^ ^14^, ^19^ ^]^ This map (**Figure** **5**a) revealed aspects of the morphological heterogeneity of the cell that remained partially hidden in conventional AFM images (Figure 5b,c). For example, the map resolved the mesh structure of the actin filament network and the size and shape of the nucleus. It also revealed the existence of patches of different scaling modulus values. Those patches were more abundant over the nuclear region.

The aforementioned map was obtained by processing 512 × 512 FDCs with the SML. However, an identical map (not shown) in terms of spatial resolution and contrast was obtained by using a model‐fitting approach based on least squares. The difference between both approaches was the processing time needed to determine the modulus and the fluidity coefficient of a single FDC. Model fitting required about 0.1 s per curve while SML needed 0.002 s per curve (**Table** **1**). Those times accumulated to generate the map. Model fitting needed almost 9 h. Most of the computational time (8 h and 15 min) was devoted to deduce the value of the modulus for each *xy* position. In contrast, the SML regressor reduced the computational time by 50‐fold. In this case, the computational time was dominated by loading the data.

**Table 1:** Times involved in processing AFM‐nanoindentation data by SML and model fitting.

Process	SML FDC [s]	Model fitting FDC [s]	SML map	Model fitting map
Loading data	0.0063	0.0063	24 min 16 s	23 min 16 s
Preprocessing	0.0008	0.0008	3 min 34 s	3 min 24 s
Parameter inference	0.0024	0.1134	10 min 40 s	8 h 15 min 0 s
Other	–	–	1 min 19 s	1 min 40 s
Total	–	–	39 min 50 s	8 h 43 min

Table 1 summarizes the time associated with the different steps of processing AFM data. The calculations were performed on the CPU of a computer with Intel i5 (4 cores). Parallel processing was not used. Additional details on the comparison test may be found in Supporting Information.

Other AFM‐based methods were proposed to generate nanomechanical property maps of cells.^[^ ^2^, ^6^, ^7^ ^]^ Those methods would also benefit from the implementation of an SML regressor similar to the one presented here.

## 5 Conclusion

In short, we developed an SML regressor to predict the nanorheology of living cells. The SML regressor improved the throughput to process AFM data to the point that the limiting factor was no longer the determinant of the nanorheological properties. The time needed to transform force–volume data into a high‐spatial‐resolution nanomechanical map was reduced from 8 h to 40 min. For the same task, the regressor was about 50 times faster than an optimized model fitting method. We estimate that additional optimizations in loading the data and the use of parallel processing in the regressor might reduce the processing time to less than 10 min.

The regressor had four main features. First, theoretical curves were applied to train and validate the regressor. Theoretical curves simplified the training of the regressor. Second, nondimensionalization is performed over all the variables involved. This step provided generality. The same regressor might predict the properties of all available mammalian cell lines. Third, the regressor consisted of two‐nested neural networks. In this way, the optimization protocol to predict the modulus is independent of the optimization protocol to deduce the fluidity coefficient. Fourth, functional data analysis was applied to generate the indentation profiles. This feature was essential to cope with the viscoelasticity of cells. It enabled to process linear and nonlinear indentation signals alike. In addition, this feature circumvented the errors associated with the indentation signals generated by the microscope. Those errors were present in model‐fitting approaches. The regressor was validated by processing AFM curves obtained from two cell lines. In both cases, the viscoelastic properties were predicted with a relative error below 4%.

## Conflict of Interest

The authors declare no conflict of interest.

## Author Contributions

**Ricardo Garcia**: conceptualization (lead); formal analysis (equal); funding acquisition (lead); investigation (equal); methodology (supporting); supervision (lead); writing—original draft (lead); and writing—review and editing (lead). **Jaime R. Tejedor**: conceptualization (supporting); formal analysis (equal); investigation (lead); methodology (lead); writing—original draft (supporting); and writing—review and editing (supporting).

## Supporting information

Supplementary Material

## Data Availability

The data that support the findings of this study are available from the corresponding author upon reasonable request.
