# Supplementary material for: High‐Throughput Nanorheology of Living Cells Powered by Supervised Machine Learning
Source: Adv Intell Syst. 2025 Apr 15;7(8):2400867. doi: 10.1002/aisy.202400867 (PMC12370168; doi:10.1002/aisy.202400867)
Supplement: Supplementary file 1 — Supplementary Material [file AISY-7-0-s001.pdf]

Supporting Information

High-Throughput Nanorheology of Living Cells Powered by Supervised Machine Learning

*Jaime R. Tejedor, and Ricardo G. Garcia**
Instituto de Ciencia de Materiales de Madrid, CSIC
c/Sor Juana Inés de la Cruz 3, 28049 Madrid, Spain
E-mail: r.garcia@csic.es

List of contents

- **Contact Mechanics Model**
- **Indentation profiles and Data Generation**
- **Functional Regression**
- **Convolutional Neural Network**
- **Normalization and Dimensionless Parameters**
- **Evaluation Metrics**
- **Training**
- **Computational Time**
- **Figure S1. Validation of the nested FBNN**
- **Figure S2. Experimental FDCs and single power-law rheology fittings**

Contact Mechanics Model

The contact mechanics model combines the relaxation function of power-law rheology with bottom-effect corrections. To determine the mechanical or nanomechanical properties of a thin sample deposited or attached to a substrate, it is important to consider the effect of the substrate on the measurements. The substrate influence on the observables is corrected by using the so-called bottom effect corrections.[1-2] Here, we assume that the substrate is much stiffer than the sample (as is usually the case for cells). The effect of the substrate is corrected in the viscoelastic model through a series expansion in the ratio of the contact radius and the thickness of the sample. The coefficients for a conical tip are listed in the table below.

Table S1. Expansion coefficients for bottom effect correction with a conical tip of semi-angle θ.

j	α_j/c	β_j
0	1	2
1	$0.721\ b$	3
2	$0.650\ b^2$	4
3	$0.491\ b^3$	5
4	$0.225\ b^4$	6

In the table, the expansion term is defined as $b = \frac{tan\,\theta}{h}$, where h is the thickness of the cell, and the proportionality constant c is defined as $c = \frac{8\ tan\,\theta}{3\pi}$.

Indentation profiles and Data generation

The data used during training (force values and indentation profiles) were generated by introducing a sampling scheme that does not rely on collecting experimental data and performing data augmentation.

We establish the following conditions for the indentation profiles: The indentation is positive and starts and ends close to zero

$$I(0) = \epsilon I(p)\ \#(\mathrm{S1a})$$

$$I(1) = \epsilon' I(p)\ \#(\mathrm{S1b})$$

ϵ and ϵ’ are numbers close to 0. The indentation has a global maximum in the interval (0,1) which satisfies

$$I'(p) = 0 \#(\mathrm{S2a})$$

$$I(p) = 1 \#(\mathrm{S2b})$$

The indentation profile is divided into approach and retraction sections where the derivative is, respectively, positive and negative

$$\begin{matrix} I'(t) > 0 & if \quad t < p \\ I'(t) < 0 & if \quad t > p \end{matrix} \#(\mathrm{S3})$$

The above conditions for the tip’s displacement profiles are quite general. They include triangular, capped sinusoids and variations in between. In fact, Equation S1-S3 encompass all the indentation profiles used in AFM-based force-distance curve measurements. In the above expressions, time and indentation are made dimensionless so their values remain in the (0,1) interval.

We generated the indentation profiles by using basis expansion. We have chosen b-splines of degree 3 as the basis functions to generate the synthetic indentation profiles. Splines are piecewise polynomials joined in a set of points, called knots, by imposing continuity and differentiability. The degree of the polynomials determines how many times the spline can be differentiated.

The basis of b-splines is defined by the recurrence relationship

$$\begin{matrix} B_{i,0}(x) = \begin{cases} 1 & if \quad t_i \leq x < t_{i+1} \\ 0 & \text{otherwise} \end{cases} \\ B_{i,k}(x) = \frac{x - t_i}{t_{i+k} - t_i} B_{i,k-1}(x) + \frac{t_{i+k+1} - x}{t_{i+k+1} - t_{i+1}} B_{i+1,k-1}(x) \end{matrix} \#(S4)$$

Additionally, they have the following property,

$$\begin{cases} B_{i,k}(x) > 0 & if \quad t_i \leq x \leq t_{i+k+1} \\ B_{i,k}(x) = 0 & \text{otherwise} \end{cases} \#(S5)$$

where t_i denotes the knots of the splines, and k denotes the degree of the spline. In other words, the basis functions have compact support and are positive over the whole domain. We use a

degree of 3 and 40 equidistant interior knots in the interval $(0,1)$. Expanding the indentation over this basis we obtain

$$I(t) = \sum_{i=0}^{N} c_i B_{i,3}(x) \, \#(S6)$$

where N + 1= m + 4 is the number of basis functions and m is the number of knots of the splines. This particular choice of basis functions allows us to translate the restrictions in the functional space to a system of linear equations and inequalities that define a polytope in Euclidean space. Finally, this volume is sampled uniformly generating an artificial set of indentation profiles for training.

To further simplify the process of sampling and reduce the degrees of freedom we take $\epsilon = 0$ in Equation (S1a),

$$I(0) = \sum_{i=0}^{N} c_i B_{i,3}(0) = c_0 B_{0,3}(0) = 0 \, \#(S7)$$

The rest of the conditions are easier to be applied over the derivative basis,

$$I'(t) = \sum_{i=1}^{N} c_i B'_{i,3}(x) = \sum_{i,j=1}^{N} c_i M_{ij} B_{j-1,2}(x) = \sum_{i=1}^{N} d_i B_{j-1,2}(x) \, \#(S8)$$

where M_{ij} is a square matrix $(N \times N)$ that relates the subspace of derivatives with the b-spline basis of degree two. The inequalities of Equation (S3) can be easily met by taking into account that the basis is positive over the entire domain,

$$\begin{cases} I'(t) > 0 & if \quad t < p \\ I'(t) < 0 & if \quad t > p \end{cases} \Rightarrow \begin{cases} d_i > 0 & if \quad i < i^*(p) \\ d_i < 0 & if \quad i > i^*(p) \end{cases} \#(S9)$$

where i^* is an index based on the position of the maximum (p) that takes into account the compact support of the basis. Note that this solution is less general than Equation (S3). Alternatively, taking into account that the basis of derivatives is of degree 2, the roots and sign of the velocity profile can be calculated and checked for each sub interval between knots.

Finally, equations S1b and S2a can be expressed as

$$I'(p) = \sum_{i=1}^{N} d_i B_{i-1,2}(p) = 0 \quad (S10a)$$

$$I(1) - \epsilon' I(p) = \sum_{i=1}^{N} c_i [B_{i,3}(1) - \epsilon' B_{i,3}(p)] = \sum_{j,i=1}^{N} d_j M_{ji}^{-1} [B_{i,3}(1) - \epsilon' B_{i,3}(p)] = 0 \quad (S10b)$$

#

The set of equations S7, S9 and S10 form a set of linear equalities and inequalities in the coefficients $\{c_0, d_i\}_{i=1}^{N}$, however, the coefficients d_i might not be bounded. The key point for reducing the space of possible solutions to a finite volume is the fact that the restrictions imposed are scale invariant. This property implies that the sets defined as $S_{d_i} = \{\alpha\, d_i | \alpha \in \mathbb{R}\}$ form equivalence classes. This property makes possible to sample a finite volume such as $|d_i| \in (0,1)$ and collapse all the solutions to a unique representative of the class by imposing the normalization.

$$I(p) = \sum_{i=1}^{N} c_i B_{i,3}(p) = 1 \, \#(S11)$$

Note that the volume selected forms a polytope in $\mathbb{R}^N$ only for fixed values of p and ϵ'. This means that p and ϵ' are additional degrees of freedom which have to be specified. In particular, ϵ' can be chosen as zero without losing generality, and $p = 0.5$ for all the operational modes used in FDC-based AFM. However, in order to simulate noise and instrument limitations in the experimental data ϵ' was sampled in the interval (-0.005, 0.005) and p was sampled in the interval (0.4, 0.6). Finally, the volume defined by the system of equations described above is uniformly sampled using a form of rejection sampling.

The scheme for generating the indentation profiles can be summarized in the following steps,

1. Select a value of p and ϵ'. Then compute the value for the index i^*.
2. Sample all the available degrees of freedom $|d_i|$ uniformly in the interval (0,1) and assign the proper sign according to Equation (S9).
3. Solve the system of equations S10 and check that the inequalities in Equation (S9) still hold and that the solution is inside the sampling volume $|d_i| \leq 1$. If not, repeat steps 2 and 4 until a valid solution is found.
4. Find the coefficients $\{c_i\}_{i=1}^{N}$ with the inverse matrix M_{ij}^{-1} and set $c_0 = 0$.
5. Divide all the coefficients by $I(p) = \sum_{i=1}^{N} c_i B_{i,3}(p)$.

Note that step 3 deviates from a typical rejection sampling scheme. In order to make an algorithm that samples uniformly the space, it would be necessary to find a basis of vectors for the orthonormal subspace of the hyperplanes determined by the system of equations S10.

However, when the number of dimensions is high very few candidates would lie inside the volume in a rejection sampling scheme. Instead, we can solve the system of equations in a way that introduces some bias, so the sampling is not truly uniform. These indention profiles are used as initial profiles for a Markov Chain Monte Carlo (MCMC) algorithm to sample the true volume defined by condition (S3) instead of the approximate solution (S10). The MCMC is applied using the following steps,

1. Define an orthonormal basis of vectors of the space determined by the system of equations S10.
2. From an initial profile generate a new profile by adding a vector in a random direction in the basis defined in step 1.
3. Verify that the profile meets the inequalities in S3 by checking the roots and sign of the corresponding velocity profile. If the profile meets S3 accept it with probability 1 otherwise reject it.
4. Repeat steps 2 and 3 and reduce the length of the steps based on the number of iterations.
5. Find the coefficients $\{c_i\}_{i=1}^{N}$ with the inverse matrix M_{ij}^{-1} and set $c_0 = 0$.
6. Divide all the coefficients by $I(p) = \sum_{i=1}^{N} c_i B_{i,3}(p)$.

A total of 500 iterations were performed. To verify the validity of this sampling scheme, the regressor was tested with theoretical curves that follow ideal indentation profiles such as, perfect triangular signals, capped sinusoids or saw-tooth profiles were the velocity is different in approach and retraction sections. The predictions of the regressor for those profiles have very small relative errors (Figure S1).

Functional Regression and Functional Neural Networks

To adapt functional data analysis into a neural network requires to implement a functional neuron. The functional neuron replaces the discrete weights found in common neurons with a weight function. The dot product of the functional input and the weight function is implemented as

$$(f_i, g) = \int f_i(x) g(x) dx \quad \#(S12)$$

We use basis expansion to compute the gradient of the loss function with respect to the weight functions and to perform backpropagation:

$$g(x) = \sum_{i=0}^{N} g_i B_i(x) \#(S13)$$

The integral in Equation (S12) is computed using two different approaches.

In what we call a functional neural network (FNN) approach, the integrals of the functional inputs with all the basis elements are computed by

$$I_{ij} = \int f_i(x) g_j(x) dx \simeq \sum_k w_k f_i(k\Delta x) g_j(k\Delta x) \#(S14)$$

where w_k are the integral weights (trapezoidal integral), then Equation (S12) is computed as

$$(f_i, g) = \int f_i(x) g(x) dx = \sum_{j=0}^{N} I_{ij} g_j \#(S15)$$

This allows for backpropagation to be performed in the usual way.

A second approach (BFNN) is to perform basis expansion also in the functional inputs by using interpolation,

$$f_i(x) = \sum_{j=0}^{N} c_j^{(i)} B_j(x) \#(S16)$$

Given that the basis functions are known, we can compute the associated gram matrix as,

$$G_{ij} = (B_i, B_j) = \int B_i(x) B_j(x) dx \#(S17)$$

the dot product is equivalent to the discrete counterpart with the metric given by G_{ij},

$$(f_i, g) = \int f_i(x) g(x) dx = \sum_{j,k=1}^{N} c_j^{(i)} g_k G_{jk} \#(S18)$$

Treating the expansion coefficients as scalar inputs gives more freedom for training. The Gram matrix can be incorporated into the network weights. Furthermore, the coefficients can be standardized to facilitate training. This implementation requires a pre-processing step of spline interpolation. For ease of use and to avoid additional interpolations, only the indentation profile was treated in this way (FBNN).

On the other hand, the force was down sampled (or up sampled) to a fixed size using a simple linear interpolation and used as normal scalar input. Nonetheless the force is normalized using group normalization. This process avoids giving too much weight to values closer to zero if it

was normalized using standardization. The sampling size was optimized as a hyperparameter in the grid search.

Nested versions of FNN and FBNN were developed following the scheme presented in Figure 2a (main text).

Convolutional Neural Network

The implementation of the convolutional neural network is straight-forward. First a fully convolutional neural network is used to process the time series data with variable length. Then an adaptive average pool layer is used to standardized the output to a fixed size. Finally, a fully connected neural network is used to obtain the mechanical parameters.

Normalization and dimensionless parameters

All of the variables are rendered dimensionless so that the neural network validity is not limited to a range of dimensional values. This process further facilitates the application of the regressor to a wide variety of experimental conditions. To that effect, the force $F(t_i)$ is divided by its mean value and the indentation is divided by its maximum value. In addition, a standard normalization is applied to all the features so that each variable has a mean value of zero and variance of one. In the case of the functional neural network and convolutional neural network, the time series data are normalized using group normalization. In other words, by using the mean and variance of all the FDC's in the training data at all the sampled times. In the case of a single neural network the output is also standardized to facilitate convergence during training, this requires an additional step to undo the standardization of the output when validating the models.

Evaluation Metrics

The models hyperparameters were optimized using a grid search with 5-fold cross validation. The metric used for the scaling or compressive modulus is the mean absolute percentage error (MAPE) computed as

$$\text{MAPE} = \frac{100}{n}\sum_{i=1}^{n}\frac{\left|E_{true}^{i} - E_{pred}^{i}\right|}{E_{true}^{i}} \#(S19)$$

And the metric for the fluidity coefficient is the normalized root mean squared error (NRMSE) computed as:

$$\text{NRMSE} = \frac{100}{\gamma_{max} - \gamma_{min}}\sqrt{\frac{1}{n}\sum_{i=1}^{n}\left(\gamma_{true}^{i} - \gamma_{pred}^{i}\right)^{2}} \#(S20)$$

The MAPE metric cannot be applied to the fluidity coefficient because it would diverge for an elastic material (γ=0).

The results of the grid search are listed below:

Model	MAPE (%)	NRMSE (%)	MAPE (%) (HeLa cell)	NRMSE (%) (HeLa cell)	MAPE (%) (Fibroblast)	NRMSE (%) (Fibroblast)
CNN	1.51 ± 0.19	1.11 ± 0.09	9.04	3.08	7.37	3.50
FNN	1.17 ± 0.08	0.91 ± 0.07	7.82	2.97	5.88	3.11
FBNN	0.89 ± 0.05	0.64 ± 0.03	10.36	4.36	7.51	4.79
Nested FNN	1.14 ± 0.05	0.88 ± 0.03	2.89	1.89	3.84	2.08
Nested FBNN	0.78 ± 0.05	0.56 ± 0.03	2.76	1.18	3.76	2.25

The number of hyperparameter configurations tested for CNN were smaller than the ones used with the FNNs. It took more time to train the CNN. The hyperparameters considered for each of the regressor models are:

CNN: Convolutional layers architecture (from 1 up to 3 convolutional layers using kernels up to size 32), input size (down sampling the input to a fixed size or using an adaptive average pool layer after the convolutional layers), fully connected network architecture (from 1 up to 3 fully connected layers with a max size of 256 neurons) and the usage of dropout layers for regularization during training.

FNN: network architecture, number of basis functions (in functional neurons) and dropout and batch normalization layers.

BFNN: network architecture, number of basis functions and number of sampled points (for the force)

Training

A total of 100000 curves were generated. The training was implemented using minibatches of 1000 curves. The loss function was the mean squared error (MSE). The initial learning rate is set to 0.01 and a small portion of training curves (100) were saved to compute a validation score to schedule the learning rate. Due to the large size of the training curves, the validation was checked for each batch instead of each epoch and a patience of 15 was used with a reduction factor of 10. The training was finished when the learning rate was bellow 10^{-5}. The maximum number of epochs was set to 30 although typically the models converged faster (5-15 epochs). In addition, several activation functions were tested being ReLu the one with the best performance.

We summarize the key features of the nested FBNN implemented in the manuscript. It is made of two NN. The first neural network has four hidden layers with sizes (100, 200, 100, 50) and ReLu activation. The second neural network has two hidden layers with sizes (50, 50) and ReLu activation

Computational Time

The computing times of the neural network and the model fitting method based on non-linear least squares were determined by using the following Python libraries, cProfile, the function perf_counter from time and snakeviz. First the implementation of the power law rheology model coupled with Ting's model was optimized. For the approach section the integral was computed using FFT based convolution. The relaxation function for the power law model is singular for times close to zero, therefore, the first steps of the integral were computed using a change of variables,

$$s = (t - t')^{\frac{1-\gamma}{2}} \quad \text{(S21)}$$

However, the FFT convolution can still be computed making slight adaptations to the integral weights and relaxation function. The retraction section of the curve requires solving Ting's condition, and the integral cannot be computed using convolution. If N is the number of points in the force distance curve, computing the force during retraction takes $O(N^2)$ operations (both for solving Ting's condition and computing the force integral). Nonetheless, the order of magnitude typical for experimental force distance curves ($N \sim 10^3 - 10^4$) is not large enough to make the asymptotic behaviour the dominant factor. Instead, the code and algorithms used to integrate the force during retraction were optimized considering the above range of values.

The non-linear least squares algorithm was performed using curve_fit function from scipy. The free parameter computed with this algorithm was just the fluidity coefficient. The model depends linearly on the compressive modulus. This allows to solve analytically the least squares minimization problem:

$$\frac{\partial}{\partial E_0}\left[\sum_i (E_0 I(x_i;\gamma) - y_i)^2\right] = 2E_0 \sum_i I(x_i;\gamma)^2 - 2\sum_i I(x_i;\gamma) y_i = 0 \ \#(S22)$$

$$E_0 = \frac{\langle I(x_i;\gamma) y_i \rangle}{\langle I(x_i;\gamma)^2 \rangle} \ \#(S23)$$

Where $I(x_i;\gamma) = F(x_i;\gamma)/E_0$. Note that this adjustment does not affect the solution of the non-linear least squares problem for the fluidity coefficient. In particular the new function to minimize is:

$$\frac{\partial}{\partial \gamma}\left[\sum_i \left(F\left(x_i;\gamma, E_0(\gamma)\right) - y_i\right)^2\right] =$$
$$\frac{\partial}{\partial E_0}\left[\sum_i \left(F\left(x_i;\gamma, E_0(\gamma)\right) - y_i\right)^2\right]\frac{\partial E_0(\gamma)}{\partial \gamma} + \frac{\partial}{\partial \gamma}\left[\sum_i (F(x_i;\gamma, E_0) - y_i)^2\right]$$
$$= \frac{\partial}{\partial \gamma}\left[\sum_i (F(x_i;\gamma, E_0) - y_i)^2\right] = 0 \ \#(S24)$$

This process speeds up the convergence of the algorithm. Note that in the case of the nested neural networks, Equation S23 can be used to compute the compressive modulus in replacement of the second neural network.

Finally, the times required to generate a hybrid map of a HeLa cell by model fitting and supervised machine learning were compared. The comparison was done on the CPU of a computer with Intel i5 (4 cores). Parallel processing was not used except from the built-in parallelization for CPU that scipy, numpy or pytorch functions may have. The times were computed both with perf_counter and cProfile.run.

In addition, it is important to note that when using non-linear least squares the function that implements the power law rheology model was called a total of 2,899,298 times, which makes an average of 11 calls per curve. The time to evaluate the PLR model was 0.0099s on average which means that 0.1094s or 96.5% of the time to perform non-linear least squares is spent

evaluating the PLR model. This confirms that the bottleneck when fitting the force distance curves is the evaluation of the PLR model.

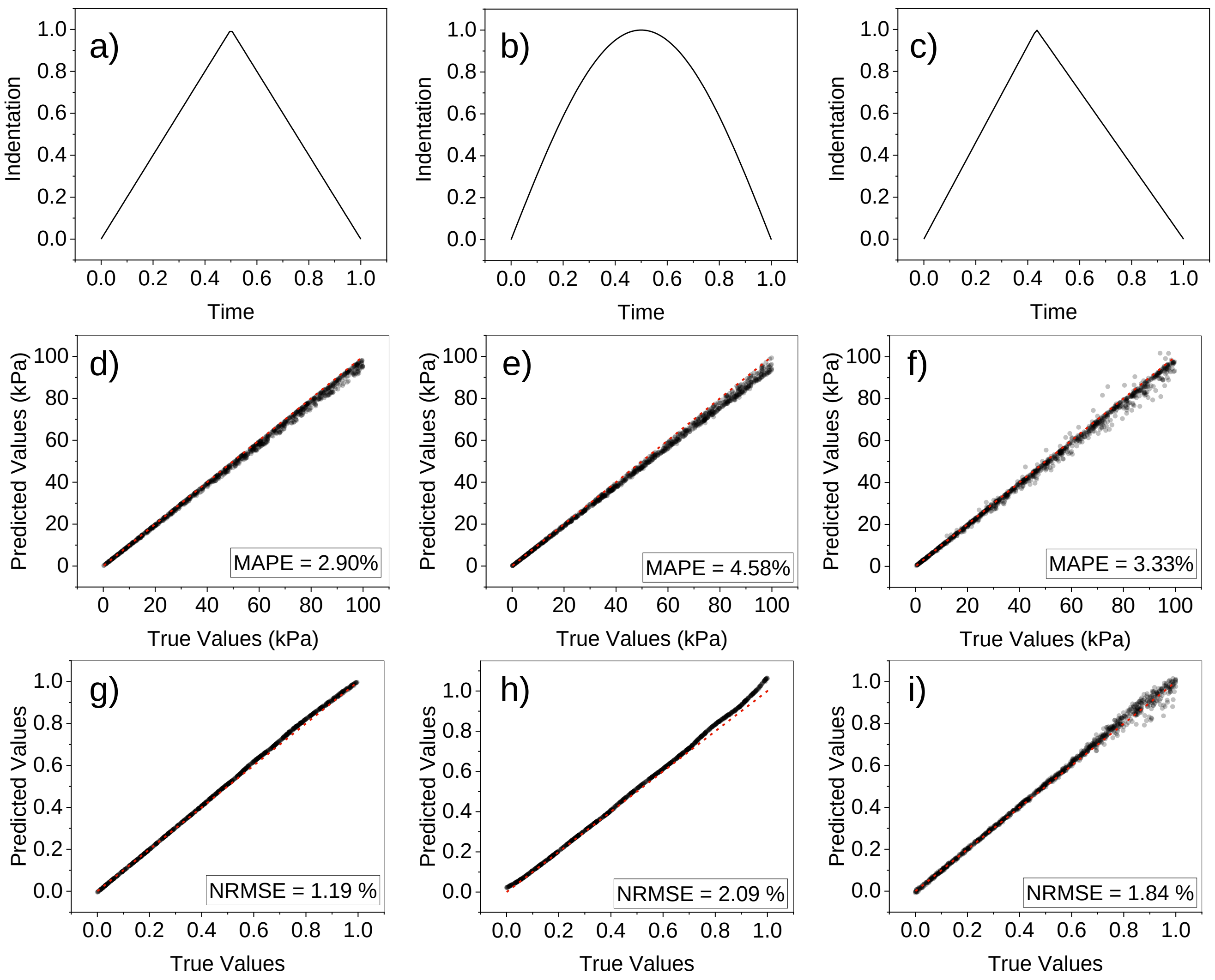

Supporting Figure S1. Validation of the nested FBNN with different theoretical curves from ideal indentation profiles found in different AFM modes. a) Example of ideal triangular indentation profile, d) and g) validation results for this type of profiles for *E* and γ respectively, b) Example of ideal capped sinusoidal indentation profile, e) and h) validation results for this type of profiles for *E* and γ respectively, c) Example of ideal uneven triangular indentation profile, f) and i) validation results for this type of profiles for *E* and γ respectively.

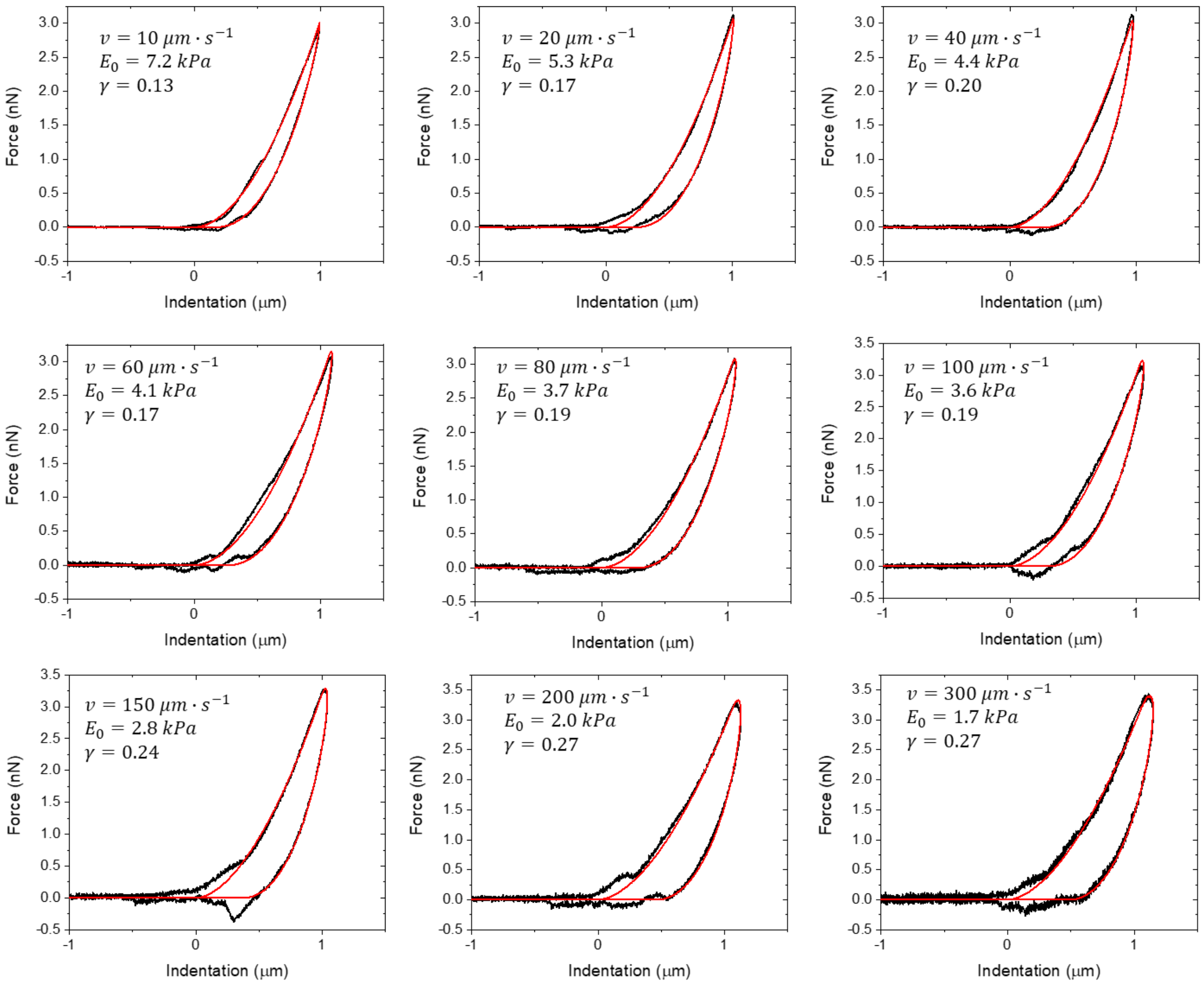

Supporting Figure S2. Experimental FDCs (black) obtained over the nucleus of a HeLa cell and the corresponding single power-law rheology fittings (red). The velocities of the indentation are included in the panels. Experimental FDCs from ref. 14 (main text).

References

1 R. Garcia. *Chem. Soc. Rev*. **2020**, 49, 5850.

2 P.D. Garcia, C.R. Guerrero, R. Garcia. *Nanoscale* **2020** 12, 9133.
